# Chronic paroxysmal hemicrania in paediatric age: report of two cases

**DOI:** 10.1007/s10194-011-0315-7

**Published:** 2011-02-22

**Authors:** Samuela Tarantino, Catello Vollono, Alessandro Capuano, Federico Vigevano, Massimiliano Valeriani

**Affiliations:** 1 Headache Center, Division of Neurology, Ospedale Pediatrico Bambino Gesù, IRCCS, Piazza Sant’Onofrio 4, 00165 Rome, Italy; 2 Center for Sensory-Motor Interaction, Aalborg University, Aalborg, Denmark

**Keywords:** Chronic paroxysmal hemicrania, Trigeminal autonomic cephalgias, Children, Indomethacin

## Abstract

Chronic paroxysmal hemicrania (CPH) is a rare primary headache syndrome, which is classified along with hemicrania continua and short-lasting unilateral neuralgiform headache attacks with conjunctival injection and tearing (SUNCT) as trigeminal autonomic cephalalgia (TACs). CPH is characterised by short-lasting (2–30 min), severe and multiple (more than 5/day) pain attacks. Headache is unilateral, and fronto-orbital-temporal pain is combined with cranial autonomic symptoms. According to the International Classification of Headache Disorders, 2nd edition, the attacks are absolutely responsive to indomethacin. CPH has been only rarely and incompletely described in the developmental age. Here, we describe two cases concerning a 7-year-old boy and a 11-year-old boy with short-lasting, recurrent headache combined with cranial autonomic features. Pain was described as excruciating, and was non-responsive to most traditional analgesic drugs. The clinical features of our children’s headache and the positive response to indomethacin led us to propose the diagnosis of CPH. Therefore, our children can be included amongst the very few cases of this trigeminal autonomic cephalgia described in the paediatric age.

## Introduction

Headache is a notable and common somatic complaint in paediatric age [1, 2]. Although migraine and tension-type headache show the highest incidence, other primary headaches are much rarer and poorly recognised, thus representing a clinical and therapeutic challenge.

Chronic paroxysmal hemicrania (CPH) is a rare and well-characterised headache, classified amongst the trigeminal autonomic cephalgias (TACs) [3]. CPH is characterised by short-lasting (2–30 min) and multiple pain attacks, with a typical attack frequency of more than 5 per day [3–6].

Pain is unilateral, located on the fronto-orbital-temporal region, it has rapid onset and it is described as excruciating. Attacks occur in combination with al least one ipsilateral autonomic symptom, such as conjunctival injection, lachrymation, miosis, ptosis, eyelid oedema, and nasal congestion or rhinorrhea [3, 7].

The standard CPH treatment is based on the use of indomethacin. The absolute pain responsiveness to indomethacin represents one of the diagnostic criteria for CPH [3] (Table 1).

**Table 1:** Diagnostic criteria for paroxysmal hemicrania (ICHD-II)

A. At least 20 attacks fulfilling criteria B–D
B. Attacks of severe unilateral supra-/orbital temporal pain lasting 2–30 min
C. Headache is accompanied by at leats 1 of the following:
1. Ipsilateral conjunctival injection and/or lachrymation
2. Ipsilateral nasal congestion and/or rhinorrhea
3. Ipsilateral eyelid oedema
4. Ipsilateral forehead and facial sweating
5. Ipsilateral miosis/ptosis
D. Attacks with a frequency of >5/day for more than half the time
E. Attacks completely prevented by therapeutic doses of indomethacin (mg kg^−1^ day^−1^)
F. Not attributed to another disorder

Because of its typical onset between 20 and 30 years of age [5, 6], CPH has been extensively studied in adult population. The disease can occur, although more rarely, also in children [8–16], as early as from the age of 3 [9, 12]. However, few reports in paediatric age fulfilled the diagnostic criteria for CPH [9, 10, 12] according to the International Classification of Headache Disorders, 2nd edition (ICHD-II) [3] (Table 2). In particular, the presence of autonomic signs and response to indomethacin has not been discussed adequately [11, 14, 15, 18]. Some unilateral short-lasting paediatric headaches without autonomic signs have been diagnosed as CPH [15, 18]. Moorjani described a case of unilateral, short-lasting and severe headache responsive to indomethacin, but the attack duration and the autonomic symptoms were not specified [18]. Despite the complete response to indomethacin is one of the diagnostic criteria [3], in some CPH children, the response to indometachin was not tested [11, 14] or this was done only in part [15]. Moreover, even one case of headache symptomatic of a cerebral infarction has been reported as CPH [16].

**Table 2:** Characteristics of previously described children with paroxysmal hemicrania

Case report	Vieira [17]	Talvik [12]^a^	Almeida [8]	Gladstein [13]	Klassen [11]	Kudrow [14]	Shabbir [15] (2 cases)	Moorjani [18]	Broeske [16]	Seidel [10]^a^	Blankenbug [9]^a^ (8 cases)
Age (years)/gender	9/M	3/F	10/F	8/M	6/M	9/M	13/F 14/F	4/F	3/F	17/M	7 mean 3F/5M
Attack duration (min)	5–30	5–15	15–40	15–30	<10	10–20	− several min − <15	Not specified	>45	10–15	15–45
Attack frequency/day	Not specified	5–20	Not specified	3	1–3 in a week	Every 1.5 h	8–9	2	2–5	10	5–20
Autonomic signs:	Absent	Yes	Yes	Yes	Yes	Yes	Not specified	Not specified	Yes	Yes	Yes
1. Lachrymation	Absent	Yes	Yes	Yes	Yes	Yes	Not specified	Not specified	Yes	No	Yes
2. Conjunctival injection	Absent	No	Yes	No	Yes	Yes	Not specified	Not specified	Yes	Yes	Yes
3. Eyelid oedema	Absent	No	Yes	No	No	No	Not specified	Not specified	No	No	Yes
4. Nasal congestion/rhinorrhea	Absent	No	No	Yes	No	Yes	Not specified	Not specified	Yes	Yes	Yes
5. Miosis/ptosis	Absent	No	No	No	Yes	Yes	Not specified	Not specified	Yes	No	Yes
Trigger	Absent	Not specified	Not specified	Not specified	Stress	Not specified	Not specified	Not specified	Not specified	Quick head movements	Stress
Symptomatic PH	No	No	No	No	No	No	No	No	Cerebral infarction	No	No
Indomethacin response	Not administered	Yes	Yes	Yes	Not administered	Not administered (baby aspirin)	Incomplete (+verapamil)	Yes	Yes	Yes	Yes

*F* female, *M* male

^**a**^CPH cases fulfilling the ICHD-II criteria

Here, we describe two children, referred to our Headache Centre, with typical CPH features and a positive response to indomethacin therapy.

## Case 1

L., a 7-year-old boy, was referred to our Headache Center because of a headache that had started 1-year earlier.

He suffered from unilateral pain, located in the right orbito-frontal region without side shift; pain intensity was excruciating, and its quality was described as throbbing. Attack duration was variable, ranging from 5 to 30 min. Headache occurred 1–3 times per day, during daytime and night-time, without circadian rhythm. Pain was combined with ipsilateral autonomic symptoms, such as conjunctival injection, eyelid oedema and rhinorrhea. During the attacks, the patient cried and found difficult to lie still showing marked agitation and restlessness. Moreover, migraine-like features, such as vomiting and photophobia, were also present. No trigger factors were detected. Psychomotor development and neurological examinations did not suggest any neurological damage. Skull CT scan, brain MRI, also including the angiographic sequences, and EEG, were normal. In addition, the blood tests and the blood pressure were normal. The boy had a positive familiar history of headache. In particular, his mother had migraine attacks when she was young.

L. was submitted to psychological screening tests (SAFA A, D, S scales) which showed normal anxiety, depression and somatization indices [19].

During the headache attack, pain intensity was not affected by most non-steroidal anti-inflammatory drugs (NSAIDs), including acetaminophen, ketoprofen, and aspirin. A prophylactic treatment with amitriptyline 16 mg/day was attempted, with no improvement in attack frequency, nor in pain intensity. When considering the headache characteristics, the clinical history and the failure of most analgesic drugs, a prophylactic treatment with indomethacin was started, at the initial dose of 25 mg/day. The attack frequency immediately showed a strong reduction, decreasing from 1 to 3 attack/day to 2–3 attacks/month. Moreover, the child’s parents were invited to use indomethacin 25 mg for each single pain attack, and they reported a strong pain relief. After 6 months, the parents discontinued the treatment voluntarily, and the headache worsened considerably. Over the following 9 months, flunarizine 5 mg/day was attempted, as suggested by another Headache Centre, without any amelioration. Conversely, a satisfactory improvement was obtained after the resumption of indomethacin 25 mg/day (attack frequency of 3–4 per months). A further reduction in headache attack frequency up to 1–2 months was obtained when topiramate was added at the dose of 45 mg/day (corresponding to 1.5 mg/kg/day). However, due to an attentive performance reduction, probably caused by topiramate, this drug was discontinued and replaced by sodium valproate at the dose of 600 mg/day. The new treatment obtained positive effects, without major adverse events. When L. was 9 years, the dose of indomethacin was increased up to 50 mg/day, with a mean attack frequency of 1 per month and without any side effect, as also confirmed by periodic blood examinations.

## Case 2

A., a 11-year-old Romanian boy, presented with a 3-year history of recurrent daily headache. He described episodes of severe throbbing pain attacks in the left fronto-orbital-temporal region. Attacks were short-lasting (ranging from 5 to 40 min), and they occurred 3–4 times per day. Attacks occurred during daytime and night-time, without nocturnal predominance, nor circadian rhythm, and most of them were combined with ipsilateral lachrymation, conjunctival injection, ptosis and nasal congestion. Pain was not accompanied by nausea, vomit nor other migraine-like features, but the child reported osmophobia during some headache attacks. Owing to his headache, the child had stopped practicing sport.

At the time of the first consultation in our centre, A. was assuming paracetamol 500 mg/day as a prophylactic treatment, without any positive effect. Moreover, his parents referred that other NSAIDs were attempted, unsuccessfully. Neurological examination was normal; brain MRI and blood tests did not show any abnormality. A. had a positive familiar history of headache, as his mother suffered from migraine attacks. No triggers factors were identified. A psychological screening was not possible, as the child could not speak Italian. Paracetamol and ibuprophen treatment were attempted without any positive effect, because pain resolved in 30–40 min also without medication.

To reduce the frequency of pain attacks, a prophylactic treatment with topiramate (50 mg/day) was started, but it proved ineffective. The clinical characteristics of our child’s headache and the failure of the traditional symptomatic and prophylactic treatments led us to consider the diagnosis of CPH. Therefore, indomethacin 50 mg/day was started, soon affecting the frequency of the attacks, which were no longer present after the second administration. A., who is continuing the indomethacin therapy at the same dose, has not suffered from any headache attack for the last 6 months. This has brought a significant improvement to his quality of life.

## Discussion

The present study describes two cases of CPH in paediatric age. Clinical symptoms and pain characteristics of our children are similar to those found in typical adult CPH. L. and A. presented with a long history of severe and unilateral pain, which occurred in the fronto-orbital region without side shift. As required by the current CPH definition [3], attacks were accompanied by at least one autonomic symptom, ipsilateral to pain. During the attacks, besides conjunctival injection, eyelid oedema and rhinorrhea, L. also presented vomiting and bilateral photophobia, which are typical migraine features, common in CPH [5, 7, 10].

The main differential diagnosis of CPH is cluster headache (CH). The relationship between CPH and CH is uncertain. In the ICHD-II, there is a considerable overlapping between the diagnostic criteria for CPH and CH, mostly concerning headache duration (from 15 to 30 min) and attack frequency (from 5 to 8), thus complicating the differential diagnosis. Moreover, also the behavioural characteristics may not be useful in distinguishing CPH from CH, since agitation, restlessness and aggressive behaviour during pain attacks, typical of CH [20], have been described also in some CPH cases [7, 12]. Pain site and associated autonomic phenomena are similar in both headaches, but the higher frequency of the attacks, their shorter duration and the absolute response to indomethacin may help in distinguishing CPH from CH [3].

Although CPH is commonly considered to prevail amongst female patients, many CPH cases in paediatric age are males [9–11, 13], as the children we described. In line with the previous cases of CPH reported during developmental age, our patients showed some atypical features, not fully meeting the ICHD-II criteria. First, although the ICHD-II criteria require an attack frequency higher than 5 attacks per day, in our patients, the attack frequency was lower. This could be in favour of a diagnosis of CH, but an attack frequency lower than the one required by the ICHD-II criteria has been described in CPH of both childhood [11, 13, 18] and adulthood [4–6]. Moreover, according to ICDH-II criteria, the attack frequency needed for diagnosing CPH should be higher than 5 per day, not constantly, but for at least half the time of observation. Second, attack duration was variable in both our children, but in A. it was sometimes longer than 30 min, which represents the maximal duration for a CPH attack, according to the ICHD-II criteria. However, several reports showed CPH patients with attack duration longer than 30 min in both adults [5, 7] and children [8, 9]. Frequency and duration of the attacks, nevertheless, are commonly different between paediatric and adult population also in more common primary headaches, such as migraine and tension-type headache. If attack duration and frequency can make the diagnosis more difficult, especially in paediatric age, the absolute response to indomethacin represents the diagnostic key for CPH in both adults and children (indotest) [3, 21]. Indeed, it is noteworthy that our children showed a dramatic response to indomethacin. In particular, L. did not respond to amitriptyline, as a prophylactic drug, nor to common NSAIDs. Moreover, after an initial response to indomethacin, the headache attacks increased their frequency after the voluntary discontinuation of the drug. They were markedly reduced when indomethacin was resumed. To avoid a prolonged treatment with a high indomethacin dose, topiramate was added, with a satisfactory clinical response, as suggested by previous studies on CPH [9, 22, 23]. Owing to cognitive adverse events, topiramate was replaced with valproate, which was effective, as well. To our knowledge, there are no studies showing the efficacy of valproate in CPH. A. did not show any improvement when treated with paracetamol and topiramate, whereas headache attacks disappeared after indomethacin was started. In none of the patients, triptans were tested, since both of them are under 12, and at their age the use of triptans is not allowed in Italy.

Long-term therapy with indomethacin is generally well tolerated [24], but adverse side effects may occur both in adulthood and childhood [9, 22]. Nevertheless both our children tolerated the therapy without any problem.

In conclusion, the characteristics of our children’s headache, particularly the positive response to indomethacin, led us to consider the diagnosis of CPH. However, the frequency and duration of our patient attacks did not fulfil the ICHD-II criteria. CPH diagnosis can be difficult in developmental age, due to the frequency and duration of attacks. Although attacks should not be longer than 30 min [3], in children’s CPH, the headache duration can exceed 40 min [8, 9, 16]. Moreover, although the ICHD-II criteria for CPH require an attack frequency higher than 5 per day, many CPH children show a lower attack frequency (2–3 per day or less) [8, 11, 13, 18]. These elements suggest that a revision of the current CPH diagnostic criteria, possibly with the inclusion of special notes for developmental age, would be necessary.
